# The hepatoprotective effects of *Pyrus biossieriana* buhse leaf extract on *tert*-butyl hydroperoxide toxicity in HepG2 cell line

**DOI:** 10.1186/s13104-021-05713-6

**Published:** 2021-08-03

**Authors:** Hamed Mir, Daniel Elieh Ali Komi, Mahdi Pouramir, Hadi Parsian, Ali Akbar Moghadamnia, Nayer Seyfizadeh, Mostafa Lakzaei

**Affiliations:** 1 Department of Nutrition, School of Allied Medical Sciences, Ahvaz Jundishapur University of Medical Sciences, Ahvaz, Iran; 2 Department of Biochemistry, School of Medicine, Jahrom University of Medical Sciences, Jahrom, Iran; 3 Cellular and Molecular Research Center, Cellular and Molecular Medicine Institute, Urmia University of Medical Sciences, Urmia, Iran; 4 Department of Clinical Biochemistry, Faculty of Medicine, Babol University of Medical Sciences, Babol, Iran; 5 Cellular and Molecular Biology Research Center, Health Research Institute, Babol University of Medical Sciences, Babol, Iran; 6 Department of Pharmacology & Toxicology, School of Medicine, Babol University of Medical Sciences, Babol, Iran; 7 Neuroscience Research Center, Tabriz University of Medical Sciences, Tabriz, Iran

**Keywords:** Antioxidant, Cytotoxicity, HepG2 cell line, *Pyrus biossieriana* Buhse, *tert*-Butyl hydroperoxide

## Abstract

**Objective:**

In present study, the effects of the leaf extract of *Pyrus biossieriana* Buhse on *tert*-Butyl hydroperoxide (t-BHP) induced toxicity in the HepG2 cell line were investigated.

**Results:**

HepG2 cells were exposed to different concentrations of both extract (1.5, 2.0, and 2.5 mg/mL) and t-BHP (100, 150, and 200 μM). The total flavonoid and phenolic contents, the cell viability, lipid peroxidation, NO generation, and the total antioxidant capacity in cell media were assessed. The amount of arbutin was estimated 12.6% of the dry weight of leaves (equivalent to 126 mg/g). Additionally, the amounts of flavonoids and phenols in extract were estimated 119 mg/g and 418 mg/g, respectively. The cells incubated with t-BHP showed a significant decrease in survival (p < 0.001). Preincubation with extract (1.5 mg/mL and 2.0 mg/mL) attenuated the t-BHP toxicity and increased the cell viability in cells exposed even to the highest concentration of t-BHP (200 μM) (p value < 0.001, and p value = 0.035) respectively. Additionally, treatment with extract reduced the cell growth suppression caused by t-BHP. The *P. biossieriana* Buhse leaf extract at concentrations of 1.5 and 2.0 mg/mL is capable of attenuating t-BHP-induced cytotoxicity in HepG2 cells.

## Introduction

The leaf extract of *Pyrus biossieriana* Buhse (a native tree that grows in the north of Iran) has been previously reported to possess anti-hyperglycemic, anti-hyperlipidemic, and antioxidant properties [1]. These leaves contain arbutin which is a glucoside of hydroquinone [2, 3]. Arbutin attenuates oxidative stress and cognitive impairment [4]. In hepatocytes, *tert*-Butyl hydroperoxide (t-BHP) is metabolized by cytochrome P-450 [5–7] and the produced free radical intermediates contribute to oxidative stress [2]. t-BHP initiates the cell death and induces mitochondrial dysfunction [8]. t-BHP mediated oxidative stress may result in DNA damage in cells through the formation of hydroxyl radicals [9, 10]. HepG2 cell line is a human hepatocellular carcinoma and nontumorigenic cell line widely used as an in vitro alternative to primary human hepatocytes in metabolism and hepatotoxicity investigations [11].

## Main text

### Materials and methods

#### Materials

HepG2 cell line was purchased from Pasteur institute-Iran. RPMI 1640, Fetal Bovine Serum (FBS), Penicillin–Streptomycin (Pen-Strep®), MTT solution, trichloroacetic acid (TCA), Arbutin (HPLC grade), and trypan blue were purchased from Sigma-Aldrich Chemical Co, UK. t-BHP was purchased from Merck Co, Germany.

#### Preparing *Pyrus biossieriana* Buhse leaf extract

The fresh leaves of *P. biossieriana* Buhse were collected from Babol city—Iran, washed, dried for 6 days, and then chopped. 400 g of powder was extracted with 2000 mL methanol (63%). The methanol portion was evaporated using a rotary evaporator. The extract was stored at − 70 °C [1, 12].

#### Determining the arbutin and flavonoid content of the extract

**Fig. 1:**
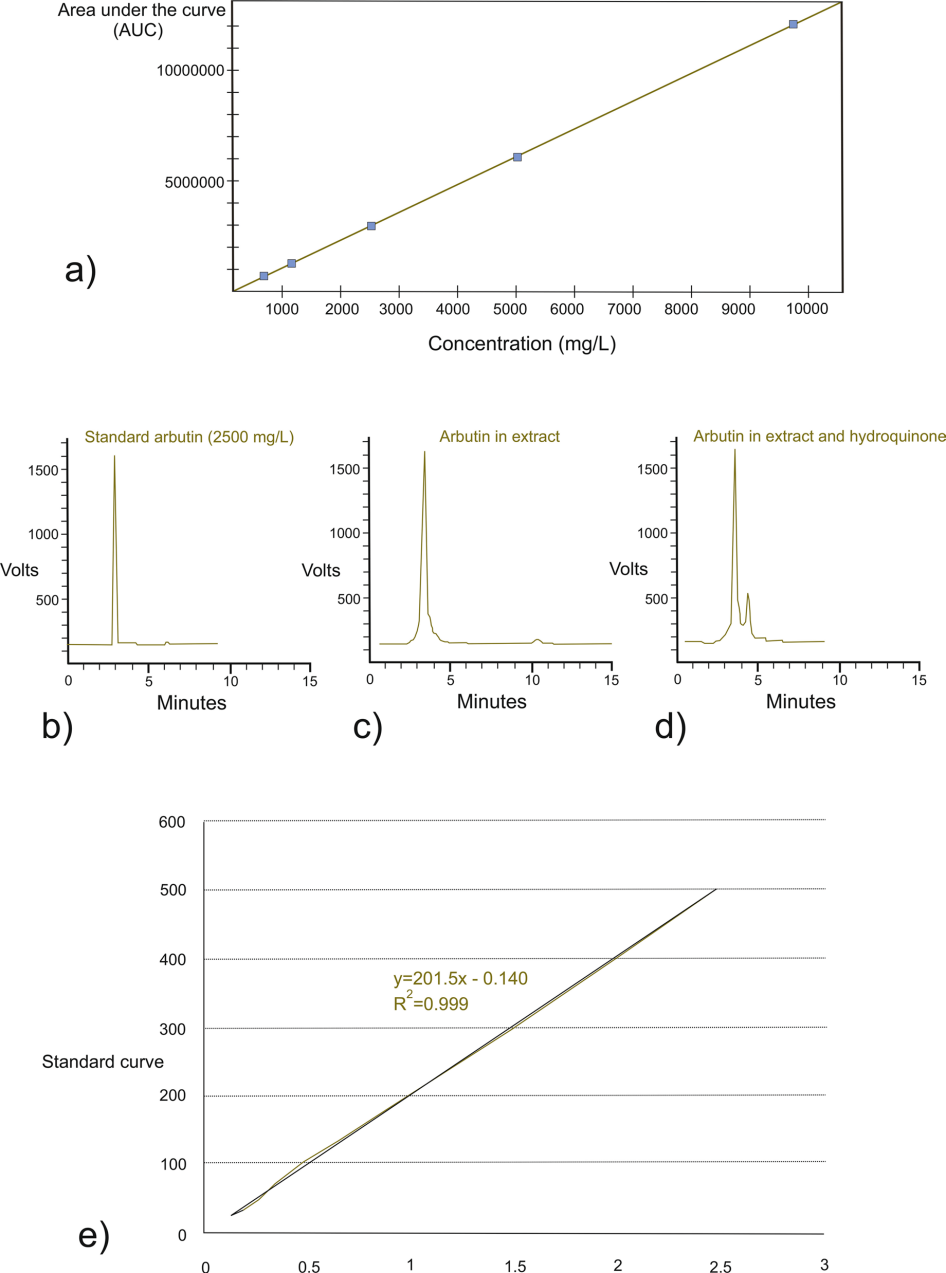
**a** Calibration curve for arbutin over the known concentration range (r = 0.99), **b** HPLC for standard arbutin (2500 mg/L) using a Eurospher C-18 column (4.6 × 250 mm), mobile phase (methanol/water 50/50; flow rate: 0.7 mL/min) detected in 286 nm wavelength, **c** HPLC to detect arbutin in extract, using the same kind of Eurospher column and detected it in 286 nm, **d** HPLC for arbutin in extract and hydroquinone (as internal standard), in similar mobile phase, flow rate, and detection wavelength, **e** standard cure for phenols was drown using gallic acid

The arbutin content of the extract was measured by HPLC. The calibration curve for arbutin over the known concentration range was linear (r = 0.99) (Fig. 1a) We performed HPLC using a Knauer Smartline Liquid Chromatography System (Knauer, Germany). Similar to our previous investigation [1], we used a Prontosil #60-5, C18 H column that was 4.6 mm in diameter and 250 mm in length. The mobile phase consisted of a 50:50 mixture of methanol and water containing 1% acetonitrile. We set the flow rate at 0.7 mL/min [1]. The percentage of arbutin recovered was determined by spiking a sample containing 7573 mg/L arbutin with an arbutin standard solution (2500 mg/L) to yield solutions with final concentrations of 7065, 6304, and 5036 mg/L. HPLC for standard arbutin (2500 mg/L) was performed using a Eurospher C-18 column (4.6 × 250 mm), mobile phase (methanol/water 50/50; flow rate: 0.7 mL/min) and was detected in 286 nm wavelength [1, 12, 13] (Fig. 1b) We then run an HPLC to detect arbutin in the extract and another HPLC for arbutin in extract and hydroquinone (as internal standard), in the same condition (Fig. 1c, d).

#### Determination of the total phenolic content

The total amount of phenolic compounds was determined by Folin-Ciocalteu reagent using the method described by Singleton and Rossi with some modifications [14]. In brief, 1 mL of extract (diluted tenfold), was mixed with 5 mL Folin-Ciocalteu reagent (diluted 1:10 with ultrapure water). After 2 min, 4 mL of sodium carbonate solution (75 g/L) was added and kept at room temperature for 2 h. The absorbance was measured at 765 nm. Total phenolic content was expressed as gallic acid equivalent (GAE) in milligrams per gram [15] (Fig. 1e).

#### Determination of the total flavonoids content

The total amount of flavonoids was determined using aluminum chloride (quercetin was used as standard). Different concentrations of standards were prepared in 60% methanol. 1 mL sample/standard was incubated in the test tube, then 1 mL of 2% aluminum chloride was added to each tube. Finally, 6 ml potassium acetate was added and OD was measured after 40 min at 415 nm [16].

#### Cell culture

HepG2 cells were plated in 24 multi-well flat-bottom culture plates, at 1.7 × 10^5^ cells per well and incubated (37 °C with 5% CO_2_ and 95% humidity) for 48 h. HepG2 cells were then divided into 16 groups each including 5 wells. The wells were exposed to different concentrations of extract (1.5 mg/mL, 2.0 mg/mL, and 2.5 mg/mL) prepared in complete media, and incubated for 24 h. After 24 h the media were removed and wells were washed twice with sterile distilled water, then different concentrations of t-BHP (0, 100 µM, 150 µM, 200 µM), were dissolved in complete media and were added to all groups, except control and incubated for 24 h [2].

#### Cell viability assay (MTT assay)

200 μL of MTT solution was added to each well and incubated at 37 °C in dark place for 4 h. Acid-isopropanol (as calibrator) (1 mL of 0.04 N HCl in isopropanol) was added to all wells and mixed thoroughly to dissolve dark blue crystals [17]. Using a spectrophotometric method, all wells were read at 570 nm as test and 630 nm as reference wavelengths [18].

#### Total antioxidant capacity (TAC)

Using ferric-reducing antioxidant power (FRAP), the total antioxidant capacity (TAC) of samples was measured. [19]. 1.5 mL of FRAP reagent was added to test tubes, incubated at 37°c for 5 min, then 50 µL of HepG2 cells media (test solution) was added to tubes, mixed thoroughly, and incubated in 37°c for 15 min. The absorbance was read at 593 [19].

#### NO generation

The Griess reagent system is based on the chemical reaction which uses sulfanilamide (SA) and *N*-1-naphthyl ethylenediamine dihydrochloride (NED) under acidic (phosphoric acid) conditions. This system detects NO_2_ in a variety of biological samples. 50 µL of samples and 50 µL of sulfanilamide were mixed gently in a microtube and incubated in dark place for 5 min. 50 µL NED was also added to each microtube. Then 50 µL Vanadium chloride (VCL3) was added to all samples, incubated at 37 °C for 45- 60 min and the absorbance of each tube was read at 540 nm [20].

#### Lipid peroxidation

Lipid peroxidation was estimated by TBARS assay, a colorimetric test for determining lipid peroxidation, based on the reaction of 2-Thiobarbituric Acid (TBA) and MDA. 0.5 mL of cell media was added to the tube containing 2 mL of reagent, mixed thoroughly, and incubated in boiling water for 15 min, allowed to reach room temperature, then centrifuged in 1500 RPM for 5 min. The OD of samples were read at 593 nm [21].

#### Statistics analysis

All groups were containing 5 wells (N = 5), except for MTT that was performed in triplicate (N = 3) to check the reproducibility. Results are presented as the mean ± standard deviation (SD). Statistical analysis was performed using one-way analysis of variance (ANOVA) with subsequent post hoc comparisons by LSD (SPSS, Ver 21.0, IBM-USA). The criterion for statistical significance is expressed as p < 0.05. The normality of data was checked using the Kolmogorov–Smirnov test.

### Results

The arbutin content of the extract was measured by HPLC (12.6% of the dry weight of the leaves). The total flavonoid and phenolic contents in the extract were 119 ± 6.93 mg/g and 418 ± 10.07 mg/g respectively. All groups that received t-BHP and extract, showed significantly reduced cell viability when compared to the control group. Incubation of cells with the extract alone partially reduced their viability in a dose-dependent manner in which incubation of cells with the extract at different concentrations of 1.5, 2.0, and 2.5 mg/mL reduced the cell viability to 94.36 ± 2.56% (p < 0.016), 81.99 ± 2.07% (p < 0.001), and 73.25 ± 1.81% (p < 0.001) respectively. In the group treated with the extract concentration of 1.5 mg/mL and 200 μM t-BHP, the cell viability decreased to 72.52 ± 1.1% (p < 0.001). The minimum rate of cell viability was observed when t-BHP at the concentration of 200 µM was added to cells pretreated with 2.5 mg/mL of extract. In this case, the cell viability dropped to 43.48 ± 3.20% (p < 0.001). Indeed, preincubation of HepG2 cells with extract (1.5 mg/mL and 2.0 mg/mL) attenuated t-BHP toxicity when the groups exposed to all concentrations of t-BHP (p values < 0.001) (Table 1) Exposure to high levels of nitrite results in toxicity, which induces the production of reactive ROS and causes oxidative stress [22]. Pretreatment of cells with the extract only, slightly increased the nitrite production [31 ± 3.33 µM in the group treated with 1.5 mg/mL concentration of extract (p = 0.09) and 31.8 ± 1.1 µM in the group treated with 2.0 mg/mL concentration of the extract when compared to the control group (p = 0.02)]. Exposure of the cells pretreated with 1.5 mg/mL of extract to different t-BHP doses (100 µM, 150 µM, 200 µM) increased the nitrite production to 40 ± 2.63, 41 ± 0.8, and 42.5 ± 1.94 µM respectively (p < 0.001). The results showed a decrease in nitrite production in t100/E1.5 treated group (40 ± 2.63 µM) and t100/E2.0 mg/mL treated group (43.7 ± 2.80 µM) (p < 0.001) (Table 2). Treatment of the cells with different concentrations of extract revealed that the extract increases the antioxidant capacity in a dose-dependent manner in which treatment of the cells with 1.5, 2.0, and 2.5 mg/mL concentrations of extract increased this parameter to 1814 ± 64.85, 2498 ± 37.83, and 3952 ± 37.83 µM (p values < 0.001). Exposure of t-BHP alone in different doses (100 µM, 150 µM, and 200 µM) reduced the antioxidant capacity in a dose-dependent manner to 140 ± 12.69 (p = 0.078), 96 ± 9.76 (p = 0.008), and 80 ± 8.8 µM (p = 0.001) respectively when compared to control group. Exposure of the cells treated with 2.0 mg/mL concentration of the extract to t-BHP concentrations (100 µM, 150 µM, 200 µM), showed a slightly decreasing trend in the antioxidant capacity (t100/E2.0, 2438 ± 32.23, p = 0.03, t150/E2.0, 2294 ± 30.81, p < 0.001, and t200/E2.0, 2234 ± 12.14, p < 0.001). A similar trend was shown when cells treated with 2.5 mg/mL concentration of the extract were exposed to 100 µM, 150 µM, and 200 µM concentrations of t-BHP in which the parameter dropped to 3832 ± 34.49, 3508 ± 23.93, and 3075 ± 97.91 µM respectively (p < 0.001). (Table 3) Lipids are the most susceptible biological molecules to the attack of ROS and RNS. Lipid peroxidation plays a role in the disturbance of fine structures, functional loss, and permeability of biomembranes and results in production of toxic products which are chemically reactive and covalently modify a variety of biomolecules including DNA bases [23]. TBA assay (TBA test) is widely used to assess the products of lipid peroxidation. From a molecular point of view, the mechanism is based on the MDA (an end product of lipid peroxidation) reaction with TBA and production of a red adduct [24]. The results of TBA test showed that t-BHP alone increased the parameter dose-dependently in which 100, 150, and 200 µM concentrations could increase TBA results to 3.17 ± 0.58, 3.86 ± 0.06, and 5.91 ± 0.18 µM (p < 0.001). Interestingly, all groups pretreated with 1.5, 2.0, and 2.5 mg/mL concentrations of the extract that were exposed to 200 µM concentration of t-BHP, showed the highest rates of lipid peroxidation [3.76 ± 0.09, 3.96 ± 0.14, and 6.23 ± 1.36 µM respectively when they were compared to corresponding groups with the same extract concentrations (p < 0.001)]. Our results showed that the extract suppresses the lipid peroxidation more effectively at 1.5 and 2.0 mg/mL concentrations (Table 4).

**Table 1:** The effect of *pyrus biossieriana* buhse leaf extract and t-BHP treatment on the cell viability of HepG2 cell line

Control 100.0 ± 1.10		p value	t100 72.86 ± 2.10		p value	t150 63.82 ± 1.7		p value
E1.5	94.36 ± 2.56	0.016	t150	63.82 ± 1.7	< 0.001	t200	47.07 ± 1.91	< 0.001
E2.0	81.99 ± 2.07	< 0.001	E1.5	94.36 ± 2.56	< 0.001	E1.5	94.36 ± 2.56	< 0.001
E2.5	73.25 ± 1.81	< 0.001	E2.0	81.99 ± 2.07	< 0.001	E2.0	81.99 ± 2.07	< 0.001
t100	72.86 ± 2.10	< 0.001	E2.5	73.25 ± 1.81	0.856	E2.5	73.25 ± 1.81	< 0.001
t150	63.82 ± 1.7	< 0.001	t100 + E1.5	85.38 ± 7.43	< 0.001	t150 + E1.5	78.94 ± 1.98	< 0.001
t200	47.07 ± 1.91	< 0.001	t100 + E2.0	76.81 ± 1.83	0.084	t150 + E2.0	68.79 ± 1.46	0.032
t100 + E1.5	85.38 ± 7.43	< 0.001	t100 + E2.5	65.49 ± 2.62	0.002	t150 + E2.5	60.01 ± 2.58	0.096

E1.5 94.36 ± 2.56		p value	E2.0 81.99 ± 2.07		p value	E2.5 73.25 ± 1.81		p value
E2.0	81.99 ± 2.07	< 0.001	E2.5	73.25 ± 1.81	< 0.001	t100	72.86 ± 2.10	0.856
E2.5	73.25 ± 1.81	< 0.001	t100 + E2.0	76.81 ± 1.83	0.026	t100 + E2.5	65.49 ± 2.62	0.001
t100 + E1.5	85.38 ± 7.43	< 0.001	t150 + E2.0	68.79 ± 1.46	< 0.001	t150 + E2.5	60.01 ± 2.58	< 0.001
t150 + E1.5	78.94 ± 1.98	< 0.001	t200 + E2.0	51.94 ± 2.72	< 0.001	t200 + E2.5	43.48 ± 3.20	< 0.001
t200 + E1.5	72.52 ± 1.10	< 0.001	E1.5	94.36 ± 2.56	< 0.001	t200 + E2.0	51.94 ± 2.72	< 0.001

The experiment was repeated five times and the average is reported with standard error for each group. Statistical analysis was performed using one-way analysis of variance (ANOVA) with subsequent post hoc comparisons by POST HOC (LSD) TEST (SPSS 21.0). (unit of measurement = percentage)

t: *tert*-Butyl hydroperoxide (t-BHP) E: *Pyrus biossieriana* Buhse leaves extract

t 100 (t-BHP, concentration: 100 µM), t 150 (t-BHP, concentration: 150 µM), t 200 (t-BHP, concentration: 200 µM)

**Table 2:** The effect of *pyrus biossieriana* buhse leaf extract and t-BHP treatment on nitrite production in HepG2 cell line

Control 28.20 ± 1.15		p value	t100 55 ± 0.97		p value	t150 56 ± 1.16		p value
E1.5	31 ± 3.33	0.090	t150	56 ± 1.16	0.573	t200	70 ± 5.40	< 0.001
E2.0	31.80 ± 1.1	0.020	E1.5	31 ± 3.33	< 0.001	E1.5	31 ± 3.33	< 0.001
E2.5	48.30 ± 1.90	< 0.001	E2.0	31.80 ± 1.1	< 0.001	E2.0	31.80 ± 1.1	< 0.001
t100	55 ± 0.97	< 0.001	E2.5	48.30 ± 1.90	< 0.001	E2.5	48.30 ± 1.90	< 0.001
t150	56 ± 1.16	< 0.001	t100 + E1.5	40 ± 2.63	< 0.001	t150 + E1.5	41 ± 0. 80	< 0.001
t200	70 ± 5.40	< 0.001	t100 + E2.0	43.7 ± 2.80	< 0.001	t150 + E2.0	47.40 ± 30	< 0.001
t100 + E1.5	40 ± 2.63	< 0.001	t100 + E2.5	59 ± 0.70	< 0.001	t150 + E2.5	60 ± 0.60	< 0.001

E1.5 31 ± 3.33		p value	E2.0 31.80 ± 1.1		p value	E 2.5 48.30 ± 1.90		p value
E2.0	31.80 1.1	0.507	E2.5	48.30 ± 1.90	< 0.001	t100	55 ± 0.97	< 0.001
E2.5	48.30 ± 1.90	< 0.001	t100 + E2.0	43.7 ± 2.80	< 0.001	t100 + E2.5	59 ± 0.70	< 0.001
t100 + E1.5	40 ± 2.63	< 0.001	t150 + E2.0	47.40 ± 30	< 0.001	t150 + E2.5	60 ± 0.60	< 0.001
t150 + E1.5	41 ± 0. 80	< 0.001	t200 + E2.0	50.20 ± 0.60	< 0.001	t200 + E2.5	65.6 ± 0.60	< 0.001
t200 + E1.5	42.5 ± 1.94	< 0.001	E1.5	31 ± 3.33	< 0.001	t200 + E2.0	50.20 ± 0.60	0.190

The experiment was repeated five times and the average is reported with standard error for each group. Statistical analysis was performed using one-way analysis of variance (ANOVA) with subsequent post hoc comparisons by POST HOC (LSD) TEST (SPSS 21.0). (unit of measurement = µM)

t: *tert*-Butyl hydroperoxide (t-BHP) E: *Pyrus biossieriana* Buhse leaves extract

t 100 (t-BHP, concentration: 100 µM), t 150 (t-BHP, concentration: 150 µM), t 200 (t-BHP, concentration: 200 µM)

**Table 3:** The effect of *pyrus biossieriana* buhse leaf extract and t-BHP treatment on antioxidant capacity in HepG2 cell line

Control 180.10 ± 14.87		p value	t100 140 ± 12.69		p value	t150 96 ± 9.76		p value
E1.5	1814 ± 64.85	< 0.001	t150	96 ± 9.76	0.351	t200	80 ± 8.80	0.486
E2.0	2498 ± 37.83	< 0.001	E1.5	1814 ± 64.85	< 0.001	E1.5	1814 ± 64.85	< 0.001
E2.5	3952 ± 37.83	< 0.001	E2.0	2498 ± 37.83	< 0.001	E2.0	2498 ± 37.83	< 0.001
t100	140 ± 12.69	0.078	E2.5	3952 ± 37.83	< 0.001	E2.5	3952 ± 37.83	< 0.001
t150	96 ± 9.76	0.008	t100 + E1.5	1381 ± 57.96	< 0.001	t150 + E1.5	1081 ± 87.36	< 0.001
t200	80 ± 8.80	0.001	t100 + E2.0	2438 ± 32.23	< 0.001	t150 + E2.0	2294 ± 30.81	< 0.001
t100 + E1.5	1381 ± 57.96	< 0.001	t100 + E2.5	3832 ± 34.49	0.002	t150 + E2.5	3508 ± 23.93	0.096

E1.5 1814 ± 64.85		p value	E2.0 2498 ± 37.83		p value	E2.5 3952 ± 37.83		p value
E2.0	2498 ± 37.83	< 0.001	E2.5	3952 ± 37.83	< 0.001	t100	140 ± 12.69	< 0.001
E2.5	3952 ± 37.83	< 0.001	t100 + E2.0	2438 ± 32.23	0.03	t100 + E2.5	3832 ± 34.49	< 0.001
t100 + E1.5	1381 ± 57.96	< 0.001	t150 + E2.0	2294 ± 30.81	< 0.001	t150 + E2.5	3508 ± 23.93	< 0.001
t150 + E1.5	1081 ± 87.36	< 0.001	t200 + E2.0	2234 ± 12.14	< 0.001	t200 + E2.5	3075 ± 97.91	< 0.001
t200 + E1.5	924.9 ± 33.78	< 0.001	E1.5	1814 ± 64.85	< 0.001	t200 + E2.0	2234 ± 12.14	< 0.001

The experiment was repeated five times and the average is reported with standard error for each group. Statistical analysis was performed using one-way analysis of variance (ANOVA) with subsequent post hoc comparisons by POST HOC (LSD) TEST (SPSS 21.0). (unit of measurement = µM)

t: *tert*-Butyl hydroperoxide (t-BHP) E: *Pyrus biossieriana* Buhse leaves extract

t 100 (t-BHP, concentration: 100 µM), t 150 (t-BHP, concentration: 150 µM), t 200 (t-BHP, concentration: 200 µM)

**Table 4:** The effect of *pyrus biossieriana* buhse leaf extract and t-BHP treatment on TBA equivalent (lipid peroxidation) in HepG2 cell line

Control 1.30 ± 0.04		p value	t100 3.17 ± 0.58		p value	t150 3.86 ± 0.06		p value
E1.5	1.28 ± 0.06	0.008	t150	3.86 ± 0.06	< 0.001	t200	5.9 ± 0.18	< 0.001
E2.0	2.44 ± 0. 03	< 0.001	E1.5	1.28 ± 0.06	< 0.001	E1.5	1.28 ± 0.06	< 0.001
E2.5	3.16 ± 0.07	< 0.001	E2.0	2.44 ± 0. 03	< 0.001	E2.0	2.44 ± 0. 03	< 0.001
t100	3.17 ± 0.58	< 0.001	E2.5	3.16 ± 0.07	< 0.001	E2.5	3.16 ± 0.07	< 0.001
t150	3.86 ± 0.06	< 0.001	t100 + E1.5	2.26 ± 0.03	< 0.001	t150 + E1.5	2.72 ± 0.07	< 0.001
t200	5.91 ± 0.18	< 0.001	t100 + E2.0	2.86 ± 0.03	< 0.001	t150 + E2.0	3.01 ± 0.05	< 0.001
t100 + E1.5	2.26 ± 0.03	< 0.001	t100 + E2.5	3.91 ± 0.07	< 0.001	t150 + E2.5	5.72 ± 0. 10	< 0.001

E1.5 1.28 ± 0.06		p value	E2.0 2.44 ± 0. 03		p value	E2.5 3.16 ± 0.07		p value
E2.0	2.44 ± 0. 03	< 0.001	E2.5	3.16 ± 0.07	< 0.001	t100	3.17 ± 0.58	< 0.001
E2.5	3.16 ± 0.07	< 0.001	t100 + E2.0	2.86 ± 0.03	< 0.001	t100 + E2.5	3.91 ± 0.07	< 0.001
t100 + E1.5	2.26 ± 0.03	< 0.001	t150 + E2.0	3.01 ± 0.05	< 0.001	t150 + E2.5	5.72 ± 0. 10	< 0.001
t150 + E1.5	2.72 ± 0.07	< 0.001	t200 + E2.0	3.96 ± 0.14	< 0.001	t200 + E2.5	6.23 ± 1.36	< 0.001
t200 + E1.5	3.76 ± 0.09	< 0.001	E1.5	1.28 ± 0.06	< 0.001	t200 + E2.0	3.96 ± 0.14	< 0.001

The experiment was repeated five times and the average is reported with standard error for each group. Statistical analysis was performed using one-way analysis of variance (ANOVA) with subsequent post hoc comparisons by POST HOC (LSD) TEST (SPSS 21.0). (unit of measurement = µM)

t: *tert*-Butyl hydroperoxide (t-BHP) E: *Pyrus biossieriana* Buhse leaves extract

t 100 (t-BHP, concentration: 100 µM), t 150 (t-BHP, concentration: 150 µM), t 200 (t-BHP, concentration: 200 µM)

### Discussion

In the present study, it was shown that t-BHP induced strong inhibition on cell growth, and pretreatment with *P. biossieriana* Buhse leaf extract (1.5 mg/mL and 2.0 mg/mL), significantly protected the HepG2 cells against oxidative damage. In the previous studies, the extract showed significant anti-hyperglycemic and anti-hyperlipemic activity [1]. Besides, it attenuates lipid peroxidation and NO production in t-BHP treated cells. Finally, our results showed that the groups treated with extract only but not t-BHP, had lower cell viability [E 1.5 mg/mL (p value = 0.016), E 2.0 mg/mL (p value ≤ 0.001), and E 2.5 mg/mL (P value ≤ 0.001)] when compared to the control group. We suggest investigating the effects of commercial arbutin, or other antioxidants reported in extract or the separated fractions of extract in further in vitro and in vivo investigations. The present study showed that *P. biossieriana* Buhse leaf extract attenuates t-BHP- induced cytotoxicity in HepG2 cells. Pretreatment with certain concentrations of extract protected HepG2 cells against alterations induced by t-BHP, probably through quenching radical species, reducing the rate of MDA formation, and NO production.

## Limitations

Application of an herbal extract according to its complicated chemical composition does not provide data on the synergic effect of components. In this regard, there may be some components that interfere with the desired and expected properties in certain concentrations, for instance in this experiment, application of extract in 1.5 and 2.0 mg/mL concentrations could effectively protect HepG2 cells. However, when extract was applied in a higher (2.5 mg/mL) concentration, the effect was partially suppressed. Determining the suppressing (and also those having synergistic effects) components may provide a better insight into the involved molecular mechanisms through which extract influences the biologic parameters studied in this and similar studies. According to our previous studies [2], arbutin was the main antioxidant, however, other extract components with suppressing or synergistic properties are yet to be defined. For instance, it was previously reported that benzoquinone is a chemical found in *Pyrus* family extract [25] and that it effectively induces apoptosis in hepatoma cell lines [26, 27]. We recommend pretreating the cells with optimized concentrations of arbutin with other components of the extract to assess the effects of arbutin on the parameters.

## Data Availability

The datasets used and/or analyzed during the current study available from the corresponding author on reasonable request.

## Abbreviations

FRAP: Ferric-reducing antioxidant power
HPLC: High-performance liquid chromatography
MDA: Malondialdehyde
RNS: Reactive nitrogen species
ROS: Reactive oxygen species
TAC: Total antioxidant capacity
t-BHP: *tert*-Butyl hydroperoxide
TBA: Thiobarbituric acid
